# Analysis of risk factors and development of a predictive model for IABP application in post-cardiac valve replacement patients

**DOI:** 10.3389/fsurg.2025.1728752

**Published:** 2026-01-13

**Authors:** Rukeya Hashan, Wang Zhengkai

**Affiliations:** 1 Department of Critical Care Medicine, First Afliated Hospital of Xinjiang Medical University, Urumqi, China; 2 Xinjiang Medical University, Urumqi, Xinjiang Uygur Autonomous Region, China

**Keywords:** cardiac valve replacement, intra-aortic balloon pump, LASSO regression, multivariate logistic analysis, predictive modeling, risk factors

## Abstract

**Objective:**

To identify risk factors for intra-aortic balloon pump (IABP) requirement following heart valve replacement surgery (HVRS) and to develop a predictive model.

**Methods:**

This retrospective cohort study analyzed 161 HVRS patients (October 2023 to January 2025) from the First Affiliated Hospital of Xinjiang Medical University. Patients were stratified into IABP (*n* = 58) and non-IABP (*n* = 103) groups. Independent risk factors were identified through univariate analysis, LASSO regression, and multivariate logistic regression. The cohort was randomly split into training and validation sets (7:3 ratio) for model development and internal validation. Model performance was assessed using receiver operating characteristic (ROC) curves, Hosmer-Lemeshow calibration, and decision curve analysis (DCA).

**Results:**

Significant differences were observed between groups across multiple parameters (all *P* < 0.05), including demographics, inflammatory markers, cardiac biomarkers, and echocardiographic indices. Multivariate analysis identified five independent risk factors for postoperative IABP use: age (OR = 1.138, 95% CI: 1.067–1.226), stroke volume (SV) (OR = 1.155, 95% CI: 1.060–1.296), cardiac output (CO) (OR = 5.700, 95% CI: 2.700–12.040), cardiac index (CI) (OR = 4.982, 95% CI: 2.879–10.119), and left ventricular end-systolic diameter (LVESD) (OR = 1.463, 95% CI: 1.157–1.849). The prediction model showed excellent discrimination in both the training set (AUC = 0.946, 95% CI: 0.910–0.982) and the validation set (AUC = 0.933, 95% CI: 0.876–0.990). Good calibration was indicated by Hosmer-Lemeshow test (*P* > 0.05 for both sets), and decision curve analysis confirmed the model's clinical utility.

**Conclusion:**

A model incorporating five routinely available preoperative variables effectively stratifies the risk of requiring IABP after HVRS, demonstrating strong discriminatory performance and potential clinical applicability for preoperative risk assessment.

## 1 Introduction

Heart valve disease (VHD), characterized by valvular stenosis or regurgitation, poses a significant threat to cardiovascular health. Its global prevalence is rising with population aging, with an estimated 40.5 million and 24.2 million cases of rheumatic and degenerative VHD, respectively (1, 2). The disease often progresses insidiously, and without timely intervention, can lead to heart failure, cardiogenic shock, or death (3). Heart valve replacement surgery (HVRS) is a definitive treatment for severe VHD, markedly improving patient outcomes. However, postoperative complications—including arrhythmias, bleeding, infection, thrombosis, and particularly Low Cardiac Output Syndrome (LCOS)—remain major challenges, critically impacting prognosis (4). LCOS results in systemic hypoperfusion, potentially triggering multi-organ failure and mortality, making its prevention and management a key concern following HVRS.

The intra-aortic balloon pump (IABP) serves as a vital mechanical circulatory support device, indicated for perioperative support in VHD, cardiogenic shock, refractory arrhythmias, and high-risk coronary disease (5, 6). By augmenting diastolic coronary perfusion and reducing systolic left ventricular afterload, IABP enhances cardiac output and function, playing an indispensable role in managing post-HVRS LCOS (7, 8). Nevertheless, IABP use carries risks such as limb ischemia, bleeding, infection, and thrombocytopenia, which can worsen clinical outcomes, prolong hospitalization, increase healthcare costs, and in severe cases, be life-threatening (9).

Therefore, accurately identifying patients at high risk for requiring IABP after HVRS is crucial. Early risk prediction would enable optimized preoperative planning (e.g., readiness for IABP support), proactive allocation of critical care resources, and more informed clinical decisions that balance the benefits of IABP against its potential complications. This underscores a clear need for a reliable predictive model. Consequently, this study aimed to analyze clinical data from 161 post-HVRS patients to identify risk factors for IABP use and to develop and validate a predictive model. Our goal is to provide clinicians with a scientific tool for preoperative risk assessment, ultimately aiming to optimize treatment strategies, improve patient prognosis, and enhance the quality of care.

## 2 Subjects and methods

### 2.1 Design

Retrospective analysis, using univariate analysis, LASSO regression and multifactorial logistic regression analysis to screen for independent risk factors for postoperative IABP application after following HVRS.

### 2.2 Time and place

The trial was completed from October 2023 to January 2025 in the First Affiliated Hospital of Xinjiang Medical University.

### 2.3 Subjects

The clinical history data of 161 patients who underwent HVRS after HVRS from October 2023 to January 2025 in the First Affiliated Hospital of Xinjiang Medical University were retrospectively analyzed, including 98 males and 63 females; the mean age was (53.17 ± 10.92) years. According to whether IABP was applied or not, the patients were divided into 58 cases in the IABP group and 103 cases in the non-IABP group. All patients gave informed consent to the experimental protocol, and the study was approved by the Ethics and Morality Committee of the First Affiliated Hospital of Xinjiang Medical University.

**Inclusion criteria:** (1) patients who applied IABP after HVRS; (2) age 18–70 years old; both of the above 2 criteria needed to be met to be included in this study.

**Exclusion criteria:** (1) patients younger than 18 years old; (2) patients with uncontrolled heart failure before surgery; (3) patients with severe respiratory function and liver, kidney, and other important organ insufficiency before surgery; (4) patients with incomplete clinical data; (5) patients with IABP-assisted implantation before and during surgery for various reasons; (6) regarding patients with IABP implantation within 12 h after surgery; (7) patients undergoing emergency surgery; (8) patients undergoing secondary surgery; and (9) patients undergoing HVRS in combination with other cardiac surgery during the same period.

This 12 h cutoff was chosen to distinguish between patients requiring IABP for primary failure to wean from cardiopulmonary bypass or immediate postoperative cardiogenic shock (often related to surgical factors, profound myocardial stunning, or acute complications) and those developing delayed or secondary Low Cardiac Output Syndrome (LCOS) later in their clinical course. By focusing on the latter group, our model aims to predict the need for mechanical support in patients who initially achieve separation from bypass but subsequently deteriorate due to factors more closely related to their preoperative reserve, ongoing ischemia-reperfusion injury, or evolving inflammatory responses. This distinction helps to homogenize the study population for the specific pathophysiology we aim to investigate.

### 2.4 Main observational indexes

The electronic medical record management system of our hospital was used to collect patients' data, including: basic demographics [gender, age, body mass index (BMI)], histories (alcohol consumption, smoking), comorbid diseases (myocardial infarction, hypertension, diabetes mellitus), preoperative vital signs (heart rate, systolic and diastolic blood pressure), and preoperative cardiac ultrasound parameters including ejection fraction (EF), stroke volume (SV), left ventricular end-diastolic diameter (LVEDD), left ventricular end-systolic diameter (LVESD), fractional shortening (FS), cardiac output (CO), cardiac index (CI), left atrial diameter, right atrial diameter, right ventricular diameter, interventricular septal thickness, left ventricular posterior wall diameter, pulmonary artery internal diameter, and right ventricular outflow tract internal diameter. Preoperative laboratory tests included hemoglobin (HGB), platelet count (PLT), albumin, aspartate aminotransferase (AST), alanine aminotransferase (ALT), total bilirubin, prothrombin time (PT), activated partial thromboplastin time (APTT), fibrinogen, urea, creatinine, N-terminal pro-B-type natriuretic peptide (NT-proBNP), troponin I, myoglobin, C-reactive protein (CRP), interleukin-6 (IL-6), and procalcitonin (PCT). Baseline information such as intraoperative blood loss was also recorded.

### 2.5 Indications for postoperative IABP application

Routine respiratory and circulatory monitoring and treatment were given after surgery, and IABP assistance was given when the patient still showed any one of the following manifestations after 12 h after surgery. Dopamine dosage >10 μg.kg^−1^.min^−1^, progressive decrease in blood pressure with the simultaneous use of two or more vasoactive drugs; postoperative cardiac output <2.0 L.m^−1^.min^−1^; mean arterial pressure <50 mm Hg (1 mmHg = 0.133 kPa), left atrial pressure >20 mm Hg, central venous pressure >15 mmHg; urine output <0.5 mL.m^−1^.min^−1^. 0.5 mL.kg^−1^.h^−1^; poor peripheral circulation, cold hands and feet; depression, inadequate tissue oxygen supply, marked decrease in arterial oxygen saturation, and persistent rise in lactate; uncontrollable malignant arrhythmias.

### 2.6 Statistical methods

SPSS 26.0 and R-studio (4.3.2) software were used for statistical analysis. For quantitative data obeying normal distribution, the data were expressed as x ± s, and vice versa, the median (quartile), and the qualitative data were expressed as frequency and constitutive ratio; for the comparison between the two groups, the independent samples *t*-test or Mann–Whitney *U*-test was used for quantitative data, and the chi-square test or Fisher's exact test was used for qualitative data. The dataset was randomly divided into a training set and a validation set according to the ratio of 7:3, and the prediction model was established using the training set, and the model efficacy was further validated on the validation set. A one-way logistic regression analysis was used to compare the differences in various factors between the case group and the control group, and to initially screen out the factors that might be associated with the application of IABP after heart valve replacement. These factors were then subjected to LASSO regression using the “glmnet” toolkit of the R software to further screen and optimize the variables, and the resulting best-fit factors (lambda.1se) were incorporated into the multifactorial logistic regression analysis to determine the factors associated with the application of IABP after heart valve replacement. Predictive ability and clinical application value were evaluated. A difference of *P* < 0.05 was considered significant.

## 3 Results

### 3.1 Analysis of the number of participants

161 postoperative HVRS patients were included, divided into 2 groups according to whether or not IABP was applied during hospitalization, 58 cases in the IABP group and 103 cases in the non-IABP group, all of which entered the outcome analysis without dropout data.

### 3.2 Grouping flowchart

**Figure 1:**
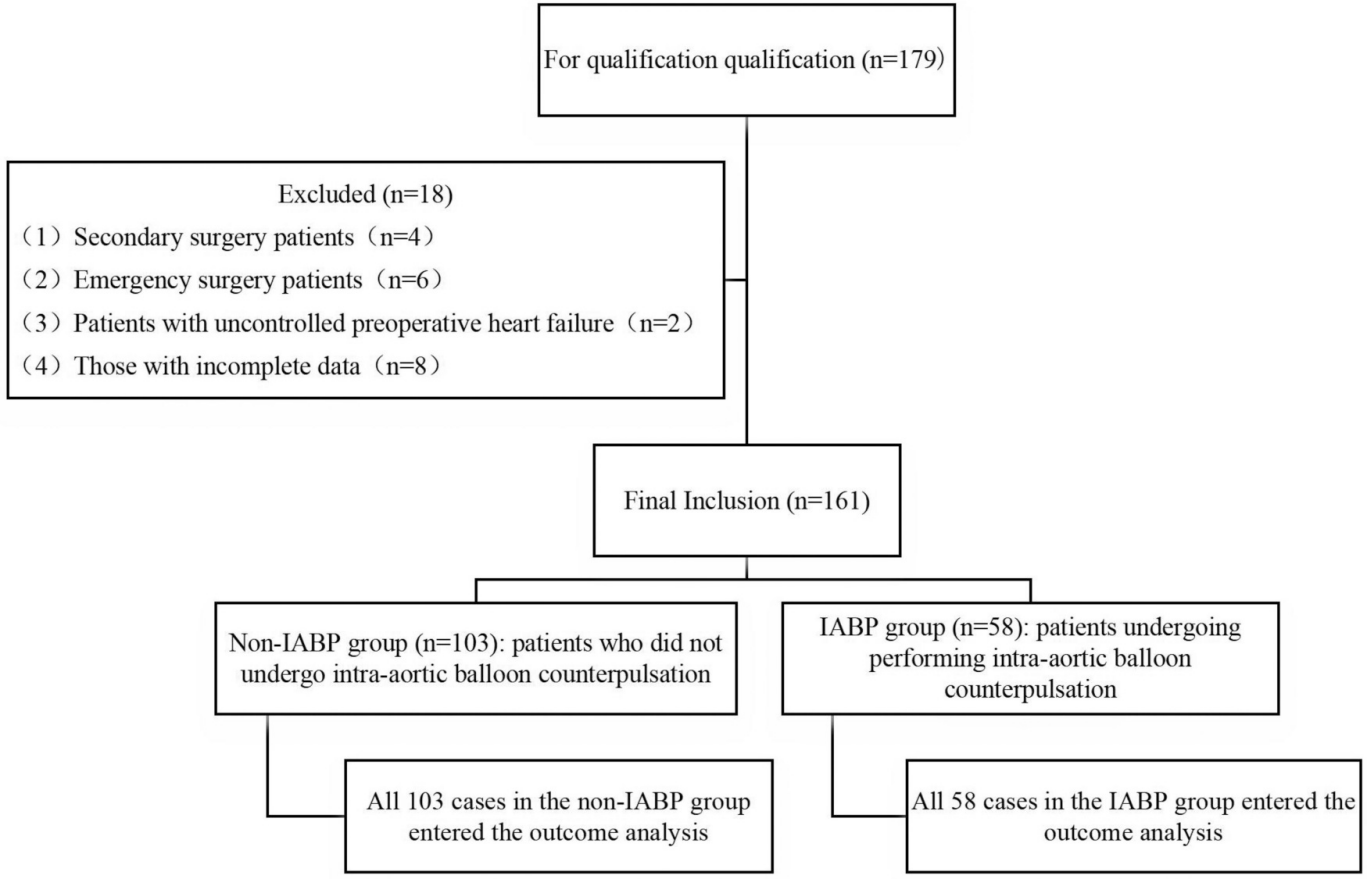
Flow chart of patient assignment.

The grouping flowcharts of the two groups are shown in Figure 1.

### 3.3 Univariate analysis

Univariate analysis showed that the IABP group compared with the non-IABP group in terms of patients' age, intraoperative bleeding, CRP, IL6, PCT, creatinine value, total bilirubin, albumin, BNP, troponin I, myoglobin, fibrinogen, PLT, FS, EF, CI, LV internal diameter, LVEDD, LVESD, ventricular septum thickness, posterior wall diameter, and RV outflow tract diameter. diameter, right ventricular outflow tract inner diameter, right ventricular inner diameter, right atrial inner diameter, pulmonary artery inner diameter, hypertension, and coronary artery disease were significantly different (*P* < 0.05). See Table 1.

**Table 1:** Basic characteristics and differential analysis.

Index	Total (*n* = 161)	no IABP groups (*n* = 103)	IABP groups (*n* = 58)	statistic	*P*
Gender				*χ*² = 1.155	0.282
Female	63 (39.1)	44 (69.80)	19 (30.20)		
Male	98 (60.90)	59 (60.20)	39 (39.80)		
Smoking status				χ² = 0.372	0.542
No	119 (73.90)	74 (62.20)	45 (37.80)		
Yes	42 (26.10)	29 (69.00)	13 (31.00)		
Alcohol consumption				χ² = 0.036	0.849
No	148 (91.90)	95 (64.20)	53 (35.80)		
Yes	13 (8.10)	8 (61.50)	5 (38.50)		
Hypertension				χ² = 5.463	0.019
No	105 (65.20)	74 (70.50)	31 (29.50)		
Yes	56 (34.80)	29 (51.80)	27 (48.20)		
Diabetes mellitus				χ² = 0.373	0.542
No	151 (93.80)	98 (64.90)	53 (35.10)		
Yes	10 (6.20)	5 (50.00)	5 (50.00)		
Coronary artery disease				χ² = 7.487	0.006
No	135 (83.90)	93 (68.90)	42 (31.1)		
Yes	26 (16.10)	10 (38.5)	16 (61.5)		
Myocardial infarction				χ² = 0.000	1.000
No	159 (98.80)	102 (64.20)	57 (35.80)		
Yes	2 (1.20)	1 (50.00)	1 (50.00)		
Age, Mean ± SD	53.17 ± 10.92	48.86 ± 10.80	56.20 ± 9.93	t = −5.542	<0.001
BMI, M (Q₁, Q₃)	25.00 (22.00, 28.00)	25.00 (22.00, 28.00)	25.00 (22.00, 28.00)	Z = 0.486	0.626
Preoperative heart rate, M (Q₁, Q₃)	80.00 (71.00, 89.00)	78.00 (70.00, 86.00)	80.00 (72.00, 90.00)	Z = −1.475	0.140
Preoperative systolic BP, M (Q₁, Q₃)	121.00 (110.00, 136.00)	120.00 (112.00, 135.00)	121.00 (110.00, 136.00)	Z = 0.040	0.968
Preoperative diastolic BP, Mean ± SD	72.47 ± 12.66	74.29 ± 12.28	71.18 ± 12.77	t = 1.925	0.055
Intraoperative blood loss, M (Q₁, Q₃)	500.00 (400.00, 600.00)	500.00 (400.00, 500.00)	500.00 (400.00, 800.00)	Z = −3.692	<0.001
CRP, M (Q₁, Q₃)	5.50 (5.00, 10.10)	5.00 (5.00, 6.20)	7.10 (5.00, 13.20)	Z = −4.328	<0.001
IL-6, M (Q₁, Q₃)	7.07 (2.94, 21.30)	3.70 (2.38,7.72)	11.36 (5.22, 73.80)	Z = −6.853	<0.001
PCT, M (Q₁, Q₃)	0.05 (0.03,0.08)	0.04 (0.03, 0.06)	0.05 (0.03, 0.16)	Z = −3.025	0.002
Serum urea, M (Q₁, Q₃)	6.63 (5.46, 8.25)	6.41 (5.05, 7.90)	7.00 (5.68, 8.39)	Z = −1.672	0.095
Creatinine, M (Q₁, Q₃)	71.87 (59.24, 86.52)	67.06 (58.39, 79.79)	78.08 (61.50, 91.08)	Z = −2.984	0.003
Total bilirubin, M (Q₁, Q₃)	15.77 (11.76, 22.72)	14.830 (11.08, 20.27)	16.370 (12.51, 24.27)	Z = −2.230	0.026
Albumin, Mean ± SD	37.73 ± 3.83	38.68 ± 3.40	37.06 ± 3.97	t = 3.457	<0.001
AST, M (Q₁, Q₃)	30.52 (24.32, 39.68)	31.29 (25.86, 40.60)	29.070 (23.44, 38.56)	Z = 1.961	0.050
ALT, M (Q₁, Q₃)	25.10 (18.53, 38.13)	24.70 (19.33, 38.13)	25.57 (17.46, 38.00)	Z = 0.518	0.605
NT-proBNP, M (Q₁, Q₃)	742.00 (257.00, 1820.00)	418.00 (126.00, 1,290.00)	988.00 (404.00, 1,970.00)	Z = −4.097	<0.001
Troponin-I, M (Q₁, Q₃)	0.01 (0.01,0.09)	0.01 (0.01,0.01)	0.036 (0.01,2.20)	Z = −7.712	<0.001
Myoglobin, M (Q₁, Q₃)	30.07 (20.96, 59.60)	24.710 (19.20, 33.16)	36.07 (23.72, 292.80)	Z = −5.423	<0.001
PT, M (Q₁, Q₃)	12.20 (11.50, 13.10)	12.10 (11.50, 12.80)	12.30 (11.50, 13.20)	Z = −1.166	0.244
Fibrinogen, M (Q₁, Q₃)	2.86 (2.50, 3.36)	2.75 (2.38, 3.07)	2.98 (2.61, 3.55)	Z = −3.721	<0.001
APTT, M (Q₁, Q₃)	31.90 (29.80, 34.10)	31.70 (29.80, 33.70)	32.10 (30.00, 34.20)	Z = −0.685	0.494
HGB, M (Q₁, Q₃)	136.00 (122.00, 103.00)	141.00 (125.00, 150.00)	133.00 (121.00, 147.00)	Z = 1.851	0.064
PLT, M (Q₁, Q₃)	207.00 (173.00, 255.00)	218.00 (189.00, 256.00)	202.00 (163.00, 247.00)	Z = 2.437	0.015
FS, M (Q₁, Q₃)	32.08 (29.17, 34.00)	33.33 (30.91, 34.62)	31.11 (25.00, 33.33)	Z = 4.705	<0.001
EF, M (Q₁, Q₃)	59.88 (55.16, 62.41)	61.29 (57.98, 63.35)	58.00 (48.08, 61.68)	Z = 4.805	<0.001
SV, M (Q₁, Q₃)	96.14 (69.63, 122.88)	85.00 (64.52, 116.93)	99.00 (70.23, 126.27)	Z = −1.879	<0.001
CO, M (Q₁, Q₃)	7.69 (5.49, 10.05)	6.61 (5.20, 9.21)	8.36 (5.12, 10.59)	Z = −1.730	<0.001
CI, M (Q₁, Q₃)	5.26 (3.76, 7.57)	3.99 (3.08, 5.22)	6.88 (4.85, 9.09)	Z = −8.138	<0.001
Left atrial diameter, M (Q₁, Q₃)	45.00 (40.00, 52.00)	44.00 (39.00, 50.00)	47.00 (41.00, 53.00)	Z = −2.534	0.011
LVEDD, M (Q₁, Q₃)	58.00 (49.00, 66.00)	55.00 (48.00, 61.00)	60.00 (51.00, 70.00)	Z = −3.093	0.002
LVESD, M (Q₁, Q₃)	39.00 (33.00, 47.00)	36.00 (32.00, 42.00)	41.00 (34.00, 50.00)	Z = −3.890	<0.001
Interventricular septum thickness, M (Q₁, Q₃)	9.00 (9.00, 10.00)	9.00 (9.00, 10.00)	9.00 (9.00, 11.00)	Z = −2.857	0.003
LV posterior wall diameter, M (Q₁, Q₃)	9.00 (9.00, 10.00)	9.00 (9.00, 10.00)	9.00 (9.00, 10.00)	Z = −2.347	0.013
Right ventricular outflow tract, M (Q₁, Q₃)	29.00 (27.00, 30.00)	28.00 (27.00, 30.00)	30.00 (28.00, 31.00)	Z = −3.442	<0.001
Right ventricular diameter, M (Q₁, Q₃)	19.00 (18.00, 20.00)	19.00 (18.00, 20.00)	20.00 (18.00, 21.00)	Z = −2.803	0.004
Right atrial diameter, M (Q₁, Q₃)	36.00 (34.00, 43.00)	34.00 (32.00, 38.00)	37.00 (34.00, 45.00)	Z = −4.109	<0.001
Pulmonary artery diameter, M (Q₁, Q₃)	25.00(23.00, 30.00)	24.00(23.00, 26.00)	27.00(24.00, 30.00)	Z = −4.455	<0.001

Data are presented as mean ± standard deviation (Mean ± SD) or median (interquartile range) [M (Q₁, Q₃)]. Units for the respective indices are as follows: age (years); body mass index (BMI, kg/m^2^); preoperative heart rate (beats per minute); preoperative systolic and diastolic blood pressure (mmHg); intraoperative blood loss (mL); C-reactive protein (CRP, mg/L); interleukin-6 (IL-6, pg/mL); procalcitonin (PCT, ng/mL); serum urea (mmol/L) and creatinine (*μ*mol/L); total bilirubin (μmol/L) and albumin (g/L); aspartate aminotransferase (AST) and alanine aminotransferase (ALT, U/L); N-terminal pro-B-type natriuretic peptide (NT-proBNP, pg/mL); troponin-I (μg/L); myoglobin (μg/L); prothrombin time (PT, seconds) and activated partial thromboplastin time (APTT, seconds); fibrinogen (g/L); hemoglobin (HGB, g/L); platelet count (PLT, ×10⁹/L); fractional shortening (FS, %) and ejection fraction (EF, %); stroke volume (SV, mL); cardiac output (CO, L/min); cardiac index (CI, L/min/m²); left atrial diameter, left ventricular end-diastolic diameter (LVEDD), left ventricular end-systolic diameter (LVESD), interventricular septal thickness, left ventricular posterior wall thickness, right ventricular outflow tract diameter, right ventricular diameter, right atrial diameter, and pulmonary artery diameter (all in mm).

LVESD, LV end-systolic diameter; LVEDD, LV end-diastolic diameter; CI, cardiac index; CO, cardiac output; SV, stroke volume; EF, ejection fraction; FS, fractional shortening; PLTc, platelet count; HGB, hemoglobin; APTT, activated partial thromboplastin time; PT, prothrombin time; ALT, alanine aminotransferase; AST, aspartate aminotransferase; PCT, procalcitonin; IL-6, interleukin-6; CRP, C-reactive protein; BMI, body mass index.

### 3.4 LASSO regression results

**Figure 2:**
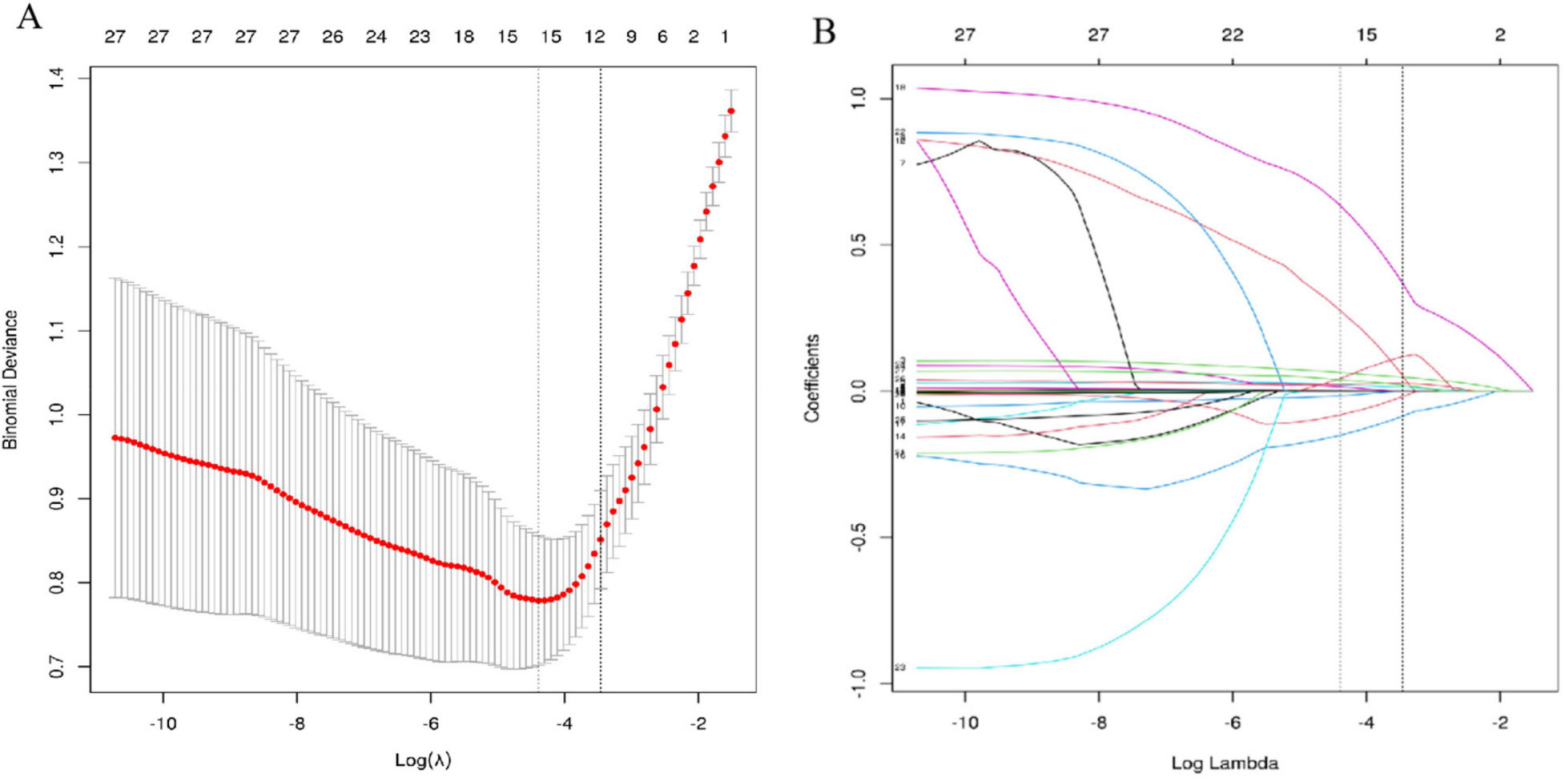
The selection of variables was optimized by LASSO regression. **(A)** is the LASSO cross-validation curve, and the best *λ* value is determined by 10-fold cross-validation; **(B)** is the path diagram of the LASSO coefficient.

To address multicollinearity and optimize variable selection, LASSO regression was applied to the variables with *P* < 0.05 from the univariate analysis. Using 10-fold cross-validation (optimal *λ* = 0.0255), the LASSO algorithm identified 27 non-zero coefficient variables for further analysis (Figure 2).

### 3.5 Multifactorial logistic regression analysis

The 27 variables selected by LASSO were entered into a multivariate logistic regression model. Five factors emerged as independent predictors for IABP use after HVRS (Table 2): age (OR = 1.138, 95% CI: 1.067–1.226), SV(OR = 1.155, 95% CI: 1.060–1.296), CO (OR = 5.700, 95% CI: 2.700–12.040), CI(OR = 4.982, 95% CI: 2.879–10.119), and LVESD (OR = 1.463, 95% CI: 1.157–1.849).

**Table 2:** Results of the multivariate logistic regression.

Predictor	*β*	S.E	Z	P	OR (95% CI)
Age	0.129	0.035	3.705	0.000	1.138 (1.067–1.226)
Intraoperative blood loss	−0.001	0.001	−1.276	0.202	0.999 (0.996–1.001)
CRP	0.044	0.035	1.284	0.199	1.045 (0.99–1.127)
IL-6	0.013	0.007	1.815	0.070	1.013 (0.999–1.032)
Albumin	−0.097	0.072	−1.339	0.181	0.908 (0.785–1.045)
NT-proBNP	0.0	0.0	1.413	0.158	1.000 (1.000–1.001)
Myoglobin	0.005	0.005	1.173	0.241	1.005 (1.000–1.017)
Fibrinogen	−0.514	0.32	−1.604	0.109	0.598 (0.334–1.290)
PLT	0.004	0.004	1.011	0.312	1.004 (0.996–1.012)
FS	0.837	0.893	0.937	0.349	2.309 (0.414–13.567)
EF	−1.014	0.631	−1.607	0.108	0.363 (0.099–1.203)
SV	0.144	0.046	3.133	0.002	1.155 (1.060–1.296)
CO	1.740	0.381	4.564	0.000	5.700 (2.700–12.040)
CI	1.606	0.319	5.036	0.000	4.982 (2.879–10.119)
Left atrial diameter	−0.01	0.036	−0.269	0.788	0.990 (0.922–1.064)
LVEDD	−0.14	0.164	−0.857	0.392	0.869 (0.518–1.119)
LVESD	−0.373	0.17	−2.19	0.001	1.463 (1.157–1.849)
Right ventricular outflow tract	0.161	0.11	1.466	0.143	1.175 (0.928–1.456)
Right ventricular diameter	−0.227	0.124	−1.825	0.068	0.797 (0.629–1.055)
Right atrial diameter	0.051	0.046	1.095	0.273	1.052 (0.96–1.153)
Pulmonary artery diameter	0.093	0.053	1.737	0.082	1.097 (0.986–1.225)
**Hypertension**					
No					
Yes	−0.115	0.585	−0.197	0.844	0.891 (0.278–2.809)
**Coronary artery disease**					
No					
Yes	0.785	0.684	1.147	0.161	2.192(0.561–8.452)

LVESD, LV end-systolic diameter; LVEDD, LV end-diastolic diameter; CI, cardiac index; CO, cardiac output; SV, stroke volume; EF, ejection fraction; FS, fractional shortening; PLT, platelet count; CRP, C-reactive protein; IL-6, interleukin-6.

### 3.6 Model performance

The prediction model demonstrated strong discriminative ability, with an area under the ROC curve (AUC) of 0.946 (95% CI: 0.910–0.982) in the training set and 0.933 (95% CI: 0.876–0.990) in the validation set. Calibration was good, as indicated by non-significant Hosmer-Lemeshow test results (training set: *P* = 0.058; validation set: *P* = 0.073). Decision curve analysis further confirmed the clinical utility of the model, showing a positive net benefit across a wide range of threshold probabilities in both datasets.

## 4 Discussion

HVRS is a key treatment for severe VHD.VHD is caused by valve stenosis or insufficient closure, and its prevalence is increasing every year with the aging of the population. Rheumatic VHD is most common in developing countries and is associated with an immune response following streptococcal infection; degenerative VHD is associated with age-related valve calcification and fibrosis and is more common in developed countries. If left untreated, VHD can lead to heart failure, arrhythmias and even death. HVRS significantly improves patient prognosis by replacing diseased valves, but postoperative complications such as arrhythmias, bleeding, infections, thrombosis, and LCOS remain important challenges (10). The core mechanism of LCOS is insufficient pumping of the heart, leading to multi-organ failure with an incidence of 3%–14% and up to 20%–30% in high-risk patients. Treatment includes positive inotropic drugs, vasoactive drugs, and mechanical circulatory assist devices (e.g., IABP and ECMO.) IABP is effective in improving cardiac function by increasing coronary artery perfusion pressure and decreasing left ventricular afterload, but its application may trigger complications such as limb ischemia, bleeding, and infection. Therefore, IABP requires strict control of the indications and timing, and close monitoring to optimize the therapeutic effect (11, 12).

IABP is a mechanical circulatory assist device inserted through the femoral artery into a balloon catheter to the descending aorta, which works by being triggered by an electrocardiogram or an arterial pressure waveform to inflate the heart during diastole to increase coronary blood flow, and deflate it during systole to decrease the left ventricular afterload, thus decreasing the burden on the heart and improving cardiac function (13, 14). Epidemiologic data show that the rate of IABP use in cardiac surgery is approximately 5%–10%, while in high-risk patients the rate can be as high as 20%. Although IABP is effective in improving cardiac function and reducing the incidence of LCOS, its use is not without risk (15, 16). Common complications include limb ischemia, bleeding, infection, and thrombocytopenia, which may aggravate the patient's condition, prolong hospitalization, and even endanger life. Therefore, the application of IABP requires strict control of the indications and timing, as well as close monitoring of the patient's relevant indexes during and after surgery, in order to minimize the risk of complications and optimize the therapeutic effect.

In this study, we explored the risk factors for postoperative application of IABP by retrospectively analyzing the clinical data of 161 postoperative patients with HVRS, divided into IABP (*n* = 58) and non-IABP (*n* = 103) groups according to whether IABP was applied or not, and constructed a columnar graphical prediction model. The results of the study showed that there were significant differences between the IABP and non-IABP groups in several baseline characteristics, including age, history of hypertension, history of coronary artery disease, intraoperative bleeding, inflammatory markers (CRP, IL-6, and PCT), renal function indexes (creatinine value), hepatic function indexes (total bilirubin, albumin), cardiac biomarkers (NT-proBNP, troponin I, myoglobin), coagulation indices (fibrinogen, PLT), and cardiac structural and functional parameters (FS, EF, CI, left atrial internal diameter, LVEDD, LVESD, septal thickness, posterior wall of the left ventricle, internal diameter of the right ventricular outflow tract, internal diameter of the right ventricle, internal diameter of the right atrium, internal diameter of the pulmonary arteries), etc., which were different and suggested that the patients in the IABP group were more complicated preoperatively and intraoperatively, the baseline risk is higher, and therefore they are more in need of IABP supportive therapy.Patients in the IABP group were older, had poorer preoperative cardiac function and more comorbidities, which were strongly associated with the development of postoperative LCOS.

We screened 27 potential predictor variables associated with IABP application by univariate and LASSO regression analyses, and further identified 5 independent risk factors including age, SV, CO, CI, and LVESD by multivariate logistic regression analysis. These results suggest that advanced age, low per-pulse output, low cardiac output, low cardiac index, and increased LV end-systolic internal diameter are significant risk factors for postoperative IABP application. Advanced age patients are usually associated with decreased vascular elasticity and reduced cardiac reserve function, which makes them more susceptible to postoperative LCOS and thus the need for IABP support. Bechri et al. (17) demonstrated that advanced age patients had a significantly increased risk of postoperative LCOS and need for IABP support, which is in line with the results of the present study, which showed that advanced age is a significant risk factor for the postoperative development of cardiac independent risk factor for postoperative IABP application. Older patients are often associated with decreased vascular elasticity, lower cardiac reserve function, and more comorbidities (e.g., hypertension, diabetes mellitus, chronic renal insufficiency, etc.), which result in poorer recovery of cardiac function after surgery. In addition, the coronary arterial reserve capacity of the older patients decreases, which makes them more susceptible to postoperative myocardial ischemia and heart failure. Therefore, elderly patients are more likely to develop LCOS postoperatively, thus requiring IABP support to improve cardiac function and systemic perfusion.

Our results are consistent with established risk factors for postoperative circulatory failure, such as advanced age and left ventricular dilation (reflected by LVESD) (17, 21), and underscore the central role of low cardiac output in LCOS pathophysiology. Unlike many existing models that depend on composite scores or isolated parameters (e.g., ejection fraction), our model integrates both structural (LVESD) and functional/volumetric (SV, CO, CI) measures—a parsimonious set particularly relevant in valvular heart disease. In conditions like valve regurgitation, preserved ejection fraction and elevated preoperative CO/CI may mask underlying contractile reserve limitation, a risk unmasked after repair. Our combined variables help quantify this dynamic. Future external validation should compare this simplified model against more complex risk scores.

Stroke Volume (SV) is the amount of blood pumped from the left ventricle into the aorta with each contraction of the heart, usually measured in milliliters (mL). It is one of the most important indexes to assess the pumping function of the heart, reflecting the efficiency and capacity of the heart per beat. Kralev et al. (18) investigators showed that reduced SV (50 mL/beat) was significantly associated with the development of postoperative LCOS, and is an important predictor of IABP application. Reduced SV directly reflects insufficient pumping function of the heart, which leads to insufficient perfusion of tissues and organs. Therefore, low SV suggests that the heart has a decreased pumping capacity per contraction and is more likely to rely on IABP to increase cardiac output and improve tissue perfusion in the postoperative period. CO is the total amount of blood pumped by the heart per minute, usually measured in liters per minute (L/min). It is a key indicator for assessing the overall pumping function of the heart and reflects the heart's ability to deliver blood to tissues and organs throughout the body per unit of time. Uhlig K et al. (19) showed that reduced CO (4 L/min) was significantly associated with the development of postoperative LCOS and was an important predictor of IABP application. Reduced CO is a direct reflection of the heart's overall pumping insufficiency, which is a central pathophysiologic mechanism of LCOS. Therefore, low CO suggests that the heart is unable to meet systemic perfusion demands and postoperative IABP support is needed to increase cardiac output and improve tissue perfusion.

CI is an important index in the assessment of cardiac function and refers to cardiac output per unit of body surface area (m^2^). Lomivorotov (20) and other researchers have shown that a reduced CI (<2.2 L/min/m^2^) is significantly associated with the development of postoperative LCOS, which is an important predictor of IABP application.CI Reduced CI reflects insufficient cardiac pumping function relative to body surface area and suggests severely impaired cardiac function. Thus, a low CI suggests inefficient cardiac pumping and a greater reliance on IABP postoperatively to improve cardiac function and systemic perfusion. LVESD is the size of the left ventricle's internal diameter at end-systole (i.e., after the heart's pumping is complete) and is usually measured by echocardiography in millimeters (mm). It is one of the most important indicators for assessing LV systolic function and the overall pumping capacity of the heart. Lang et al. (21) demonstrated that an enlarged LVESD (>40 mm) was significantly correlated with the development of postoperative LCOS, and was an important predictor of IABP application. An enlarged LVESD suggests that the LV systolic function is diminished, and the heart's pumping capacity is reduced. This study has important clinical significance. By identifying the independent risk factors for postoperative IABP application, the risk level of patients can be more accurately assessed in the preoperative period, so as to formulate a personalized treatment plan, and it can be determined in advance that those patients need the adjuvant therapy of IABP. In addition, the application of the nomogram prediction model can optimize the allocation of medical resources and improve the efficiency of medical treatment.

This study identified that both increased LVESD (a marker of ventricular dilatation and systolic dysfunction) and higher SV/CO/CI collectively predict IABP need. This pattern may reflect the unique hemodynamics in a subset of patients with valvular heart disease, such as those with significant aortic or mitral regurgitation. In these conditions, volume overload leads to left ventricular dilation (increased LVEDD and LVESD) and eccentric hypertrophy. While the EF may be preserved or even increased in the compensatory phase, leading to elevated SV and CO, the underlying systolic function per unit myocardial mass is often compromised. The enlarged LVESD signifies this maladaptive remodeling and reduced contractile reserve. Postoperatively, after valve replacement eliminates the regurgitant volume, the ventricle's inability to maintain adequate forward flow against the new afterload conditions can unmask latent systolic failure, precipitating LCOS and the need for IABP support. Thus, our model captures both the structural vulnerability (enlarged LVESD) and the potentially deceptive preoperative high-flow state (elevated CI/SV/CO), providing a more holistic risk assessment (22, 23).

**Limitations of this study:** (1) This study was a single-center retrospective study with a relatively small sample size, which may be subject to selection bias and information bias. (2) This study only focused on the application of IABP and did not consider the effects of other circulatory assist devices, e.g., extracorporeal membrane pulmonary oxygenation. (3) Although the nomogram model constructed in this study has a high degree of predictive accuracy, external validation of the model needs to be performed in a larger larger population to ensure the general applicability of the model. The present study can appropriately expand the sample size in the course of future research, change the single-center into a multicenter, prospective study, and include other circulatory assist devices for comparative analysis. (4) Fourth, while the nomogram demonstrated high discriminatory ability in both the training and validation sets (AUC > 0.93), the sensitivity in the validation set was suboptimal (0.309). This discrepancy suggests that the optimal probability cutoff value derived from the training set might not be directly transferable to the validation cohort. In clinical practice, the cutoff can and should be adjusted based on the specific clinical context—prioritizing sensitivity (to ensure no high-risk patient is missed) or specificity (to avoid unnecessary interventions). The relatively low sensitivity in our validation set may also indicate a degree of overfitting, despite our use of regularization techniques and external validation. Future studies with larger, multi-center cohorts are needed to further calibrate the model and optimize its classification thresholds. (5) the predictors in this model incorporate echocardiographic parameters, whose measurements might vary between different operators. This inter-observer variability could potentially impact the model's generalizability to some extent. (6) Fourth, our model incorporates several interrelated hemodynamic variables (SV, CO, CI). While LASSO regression was employed to mitigate multicollinearity during variable selection, residual correlations may still affect the stability and precise interpretation of individual coefficients. The primary aim was to build a robust predictive tool, and this set of variables collectively provides a strong signal for risk stratification. Fifth, although internal validation showed excellent discrimination (AUC > 0.93), the relatively modest single-center sample size increases the possibility that the model's performance is optimistic, reflecting some degree of overfitting. Therefore, the most critical next step is external validation in larger, multicenter, prospective cohorts. Such validation is essential to: (1) confirm generalizability across diverse populations and clinical settings; (2) assess the impact of inter-observer variability in echocardiographic measurements; (3) further refine variable selection among correlated parameters; and (4) optimize classification thresholds. Until such validation is completed, clinical application of this nomogram should be cautious and primarily serve as an adjunctive decision-support tool.

In conclusion, in this study, by retrospectively analyzing the clinical data of patients after HVRS, age, SV, CO, CI, and LVESD were important indicators to predict the application of IABP after HVRS. These indexes reflect the patients' cardiac functional status, which is more likely to develop LCOS and need IABP supportive therapy after surgery. Independent risk factors for postoperative application of IABP were identified, and a column-line graph prediction model containing five risk factors was constructed. The model has high predictive accuracy and clinical practicability, which can provide clinicians with scientific and reliable decision support to optimize the treatment plan and improve the prognosis of patients, and is expected to further improve the therapeutic effect and quality of life of postoperative patients with HVRS.

## Data Availability

The raw data supporting the conclusions of this article will be made available by the authors, without undue reservation.
